# Pore- and Core-Scale Recovery Performance of Consortium Bacteria from Low-Permeability Reservoir

**DOI:** 10.3390/microorganisms11112738

**Published:** 2023-11-09

**Authors:** Ziwei Bian, Zhiyong Song, Zena Zhi, Xiangchun Zhang, Yiqian Qu, Ruiyang Chai, Hanning Wu, Yifei Wu

**Affiliations:** 1 Department of Geology, State Key Laboratory of Continental Dynamics, Northwest University, Xi’an 710069, China; 202110382@stumail.nwu.edu.cn (Z.B.); zhizena@stumail.nwu.edu.cn (Z.Z.); l_spring_2004@126.com (X.Z.); quyiqian623@126.com (Y.Q.); chairuiyang2022@163.com (R.C.); 2 School of Civil and Resource Engineering, University of Science and Technology Beijing, Beijing 100083, China; songzhy232@163.com; 3 College of Food Science and Technology, Northwest University, Xi’an 710069, China

**Keywords:** microbial enhanced oil recovery, consortium bacteria, visualization, tight core, recovery efficiency

## Abstract

Performance evaluation of microorganisms that have emulsifying and degrading effects on crude oil has been extensively conducted in the laboratory. However, the ultimate goal of microbial enhanced oil recovery is field application, so the pilot simulation experiments are crucial. In this study, a micro-visualization model and the real cores were chosen to investigate the actual recovery efficiency and the mechanism of the consortium bacteria B-ALL, which has been proven to have good emulsification and degradation effects in lab studies in porous media. At the same time, the cast thin sections and rate-controlled porosimetry were combined to analyze the pore throat structure of the displacement core. It was found that the recovery efficiency was positively correlated with the microbial injection volume as well as the incubation time. For the microscopic model with high pores and high permeability, the efficiency of secondary water flooding can be increased by 44.77% after six days of incubation with two pore volume microbes. For the real tight cores, the maximum secondary water flooding efficiency under the same condition was 6.98%. Through visual modeling, microorganisms increase the oil washing efficiency mainly by emulsification and changing the wettability. The generated oil droplets will play a role in plugging and improving the wave efficiency. However, tight reservoirs have the characteristics of large pores and small throats, and curved and necking throats are developed, greatly reducing permeability. The microbial recovery efficiency was lower under shorter cultivation times. This study provides a practical basis for the application of consortium bacteria in tight oil fields to enhance recovery.

## 1. Introduction

Microbial enhanced oil recovery (MEOR) is a promising tertiary oil recovery technology based on the survival and metabolism of petroleum microorganisms in the reservoir to modify the reservoir and crude oil to enhance oil recovery [1,2]. Metabolites produced by microbes such as biosurfactants, biopolymers, and acids have been proven to reduce interfacial tension and viscosity and degrade crude oil [3,4,5]. Compared with existing tertiary oil recovery methods, MEOR has a relatively lower cost. MEOR has been proven to be economical through numerous experiments, effectively reducing costs and achieving higher recovery rates at low inputs [6,7,8]. In addition, they are environmentally friendly, and some microorganisms can even be used to remediate oil-contaminated soil and oceans [9,10]. Many scholars also obtained different strains in the oil field by various methods and confirmed the effect of strains on the recovery rate through laboratory experiments [11,12,13,14].

However, real oilfield reservoirs are high-temperature and high-pressure porous media with high non-homogeneity [15,16]. The transport of fluids and mechanisms is very complex. For China, the widely distributed low permeability and tight oil fields have more stringent reservoir conditions, and there is more remaining oil [17]. Evaluation experiments in the laboratory are not sufficient to fully simulate the actual conditions, and all extraction technologies ultimately serve the on-site extraction of oil fields. However, field tests are difficult to conduct at any time due to economic and other constraints. Therefore, conducting simulated flooding of real cores is an important way to verify the indoor results as a precursor to field experiments.

The microscopic glass etching model is a pore-scale model etched on a glass plate based on the pore throat structure of the natural core. It has the advantage of visualization, which can visually explore the distribution state of the remaining oil and its transport [15,18,19]. The simulated water flooding experiments are at the core scale, which can approach the real stratigraphic conditions to the maximum extent [20]. These two methods have been widely used in the study of seepage flow characteristics [21,22,23], but are rarely seen in MEOR studies, while visualization is important to visualize the role of metabolites and the transport of microorganisms.

Therefore, this study selected a microscopic glass etching model and the natural tight core for the MEOR simulated experiments, combined with cast thin sections and rate-controlled porosimetry injection analysis of the pore-throat structure. The crude oil flow characteristics and residual oil distribution before and after microbial action were observed, as well as the effect of microbial injection volume and cultivation time on the recovery rate. This study verifies the effect of the consortium strain on recovery and provides a realistic and reliable basis for the application of MEOR in the low-permeability and tight field.

## 2. Materials and Methods

### 2.1. Materials

The bacterial consortium was constructed by three single strains, SC4534 (2) (*Bacillus* sp.), SC4542 (*Bacillus licheniformis*), and A-3 (*Bacillus licheniformis*), isolated from a low-permeability reservoir, in a 1:1:1 ratio. All of the strains had the biosurfactant-producing ability. The characteristics and performance of this bacterial consortium in the lab have been stated in Bian et al. (2023) [24]. B-ALL could survive at 25–55.7 °C, 1–50 g/L salinity, and 5–10 alkaline environments, which means it has good tolerance. B-ALL has the best emulsification and degradation ability, with a further 23.81% reduction in degradation rate compared to the single bacteria. Crude oil (viscosity > 64,000 mPa·s) in experiments was provided by Shengli Oilfield and diluted to 70 mPa·s with kerosene.

### 2.2. Microscopic Flooding Experiments

#### 2.2.1. Apparatus

**Figure 1:**
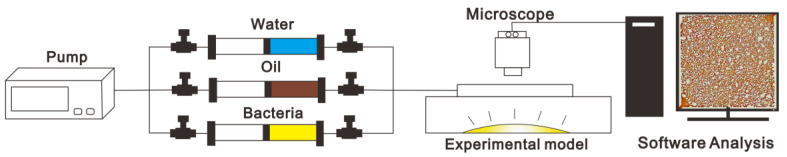
Microscopic visualization model (modified according to [28]).

The micro visualization device consists of two parts: a displacement device and an image acquisition system (Figure 1). The effective area of the model was 40 × 40 mm. The average pore throat was 35–40 μm, and the model was clamped by thick glasses [25]. The pore-throat structure of the glass etching model outlined a real sandstone core [26,27]. 

#### 2.2.2. Procedure

1. The model was vacuumed and saturated with kerosene for five hours and the vacuum was maintained overnight. Then, simulated crude oil was used to displace the kerosene and simulate the state of crude oil in the reservoir.

2. The water was injected with 20 μL/min, representing the first water flooding. The residual oil state in the pores after water flooding was carefully observed, a stereo microscope was used to take pictures (Nikon SMZ745T, Tokyo, Japan), and the proportion of crude oil was analyzed through PS (Photoshop CC 2019, Adobe, CA, USA). Then, the oil proportion with Formula (1) and water flooding efficiency was calculated with Formula (2):

(1) $$Oilproportion(P)=(S_{O}/S_{T})\times 100\%$$

(2) $$EORE(W)=((P_{I}-P_{W})/P_{I})\times 100\%$$

S_O_: The area occupied by brown (color of crude oil) in the figure; S_T_: The total area of the image; EORE(W): Enhanced oil recovery efficiency of first water flooding; P_I_: The proportion of initial crude oil in the model; P_W_: the proportion of crude oil after water flooding.

3. A total of three groups were carried out and pure water flooding was used as a control group. Microbial injection volume and cultivation time were used as variables. The first group used 1 PV (pore volume, approximately 50 μL) and incubated for 3 days after injection. The second group was infused with 2 PV and incubated for 3 days. The third group was infused with 2 PV and incubated for 6 days. The injection rate was 6 μL/min and the culture temperature was 42 °C. To maintain air tightness, the model was placed in the sink. The proportion of remaining oil after injecting microorganisms was recorded and the enhanced oil recovery efficiency of microbes (EORE(M)) was calculated. Formula (3) is as follows:

(3) $$EORE\left(M\right)=(\left(P_{W}-P_{M}\right)/P_{W})\times 100\%$$

P_M_: proportion of crude oil after microbial flooding.

4. After cultivation, a second water flooding was carried out, and the remaining oil state in the model after the second water flooding was photographed. The enhanced oil recovery efficiency of second water flooding (EORE(S)) was calculated using Formula (4). It is necessary to clean the model with kerosene and repeat step (1) after each experiment.

(4) $$EORE\left(S\right)=((P_{M}-P_{S})/P_{M})\times 100\%$$

P_S_: proportion of crude oil after second water flooding.

### 2.3. Microscopic Pore-Throat Structure Analysis

The cores in the test were Berea cores, which have been proven to have good repeatability and can reflect oil displacement characteristics in core flooding experiments.

#### 2.3.1. Casting Thin Section

The distribution characteristics of pores and throats in reservoirs were determined by the composition of rocks and the arrangement and sorting of particles. The casting thin section could effectively show the granularity, connectedness, distribution, and combination characterization of the samples [29].

Cast thin sections are made by injecting colored liquid glue into the pores of rocks, and the pores are filled with solidified hard reactants to form a rock cast. The rock thin sections were ground into thin slices to obtain a two-dimensional cross-section, and the two-dimensional images were scanned to obtain a thin-section image. The production and identification of thin film sections were carried out followed by the standards SY/T 5913-2021 [30] and SY/T 5368-2016 [31].

#### 2.3.2. Rate-Controlled Porosimetry

The reservoir space of sandstone is a complex and varied pore-throat system composed of various types of pores and throat connections. Rate-controlled mercury (RCP) can clearly characterize the differences in pore and throat structures. Combining image information (cast thin sections) can qualitatively and quantitatively reflect micro pore throat characteristics such as pore-throat size and connectivity [32,33].

The RCP was performed by a rate-controlled mercury injection instrument (ASPE-730, Willoughby, OH, USA). The injection rate was a quasi-static constant value of 5 × 10^−5^ mL/min. The experimental temperature was 25 °C and the mercury contact angle was 140°. The surface tension was 480 mN/m. The maximum injection pressure was 900 psi (6.2 Mpa), and the maximum pressure was maintained until the end of the experiment. The minimum distinguishable pore throat is 0.12 μm.

### 2.4. Core Flooding Experiments

#### 2.4.1. Apparatus

**Figure 2:**
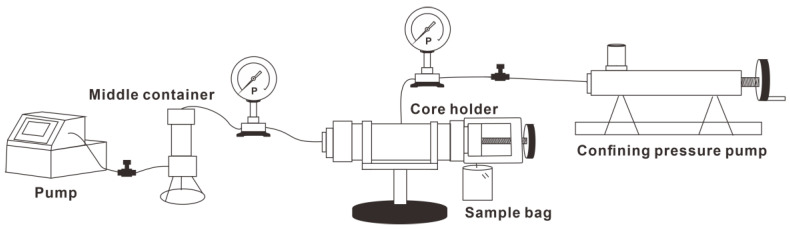
Core flooding apparatus.

The core flooding apparatus is shown in Figure 2 and the bacteria were placed in the middle container. Due to the small amount of displaced crude oil, the weighing method is used to monitor the amount of oil and water extracted. Sample bags were used to obtain the sample, and the recovery rate was calculated by weight.

#### 2.4.2. Procedure

1. The rock core was placed in a vacuum drying oven at 100 °C and vacuumed for three hours (to remove air from the pores), then quickly weighed (W_D_). The weighed rock core was placed in a beaker filled with water and vacuumed again for three hours before being left overnight. The surface moisture was dried and then weighed again (Ww). The pore volume (PV) was calculated according to formula (5), where the density of water (ρ_W_) is considered to be 1 g/cm^3^.

(5) $$PV=(W_{W}-W_{D})/\rho_{W}$$

2. The saturated water core was vacuumed at 100 °C again for three hours to restore it to its original dry state. Subsequently, it was quickly transferred to a container containing simulated oil for saturation oil, then pressurized at 13 Mpa. The rock core was placed in crude oil for two days of aging, then removed and weighed again (Wo). Then, the weight of saturated oil (W_S_) was calculated with Formula (6):

(6) $$W_{S}=W_{O}-W_{D}$$

3. A water drive experiment was conducted by injecting 1.5 PV of water at 0.04 uL/min, with a confining pressure set at 14 MPa. The extracted liquid was collected and weighed to obtain the oil weight of water flooding, and then the efficiency of water flooding through was calculated using Formula (7). Subsequently, microorganisms were injected under 13 MPa of pressure and three sets of experiments were designed, corresponding to the microscopic visualization experiment. 0.5 PV of microorganisms were injected into the B-01 core and soaked for three days; the B-02 core was injected with 1 PV microbial soaking for three days; the B-03 core was injected with 1 PV of microorganisms and soaked for six days. The temperature was 42 °C.

(7) $$Efficiency\;of\;water\;flooding=(Oil\;weight\;of\;water\;flooding/W_{S})\times 100\%$$

4. Two PV of water was injected into the core to conduct second water flooding with the same injection pressure and the oil weight of the second water flooding was weighed. The efficiency of second water flooding was calculated by following Formula (8):

(8) $$Efficiency\;of\;second\;water\;flooding=(Oilweightofsecondwaterflooding/W_{S})\times 100\%$$

5. In addition, core B-04 was directly injected with microbes (1 PV) after saturation oil and aging, and then water flooding was continued to calculate the recovery rate of direct application of microbial flooding.

## 3. Results

### 3.1. Microscopic Visualization Experiment

**Figure 3:**
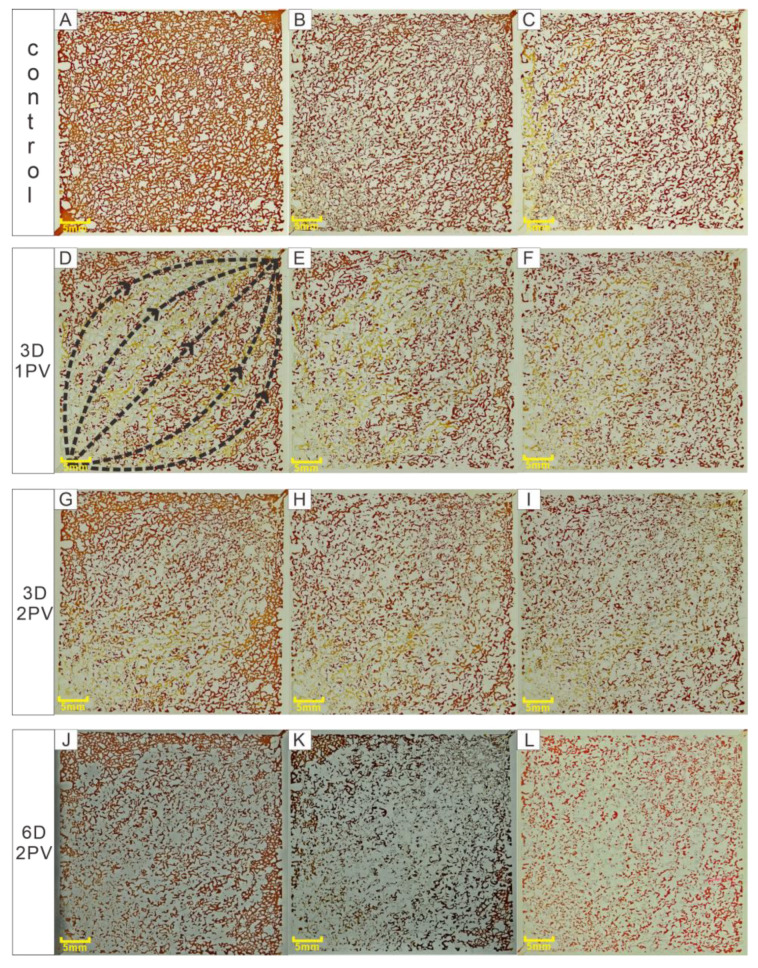
Microscopic visualization of MEOR; D is the number of culture days, and PV is the pore volume ((**A**)—Model with saturated oil; (**B**)—Primary water flooding of the control group; (**C**)—Second water flooding of the control group; (**D**,**G**,**J**)—Primary water flooding of the test group; (**E**,**H**,**K**)—Injecting microorganisms for 0 days; (**F**,**I**,**L**)—The second water flooding of the test group).

Microbial flooding tests were conducted with a microscopic glass etching model to study the effects of incubation days and microbial injection volume on recovery efficiency. The results are shown in Figure 3. The repeated utilization of the model resulted in an inconsistent proportion of oil each time, so the initial oil proportion may have a small variation (Table 1).

The water flow would quickly form a dominant channel during the water drive (Figure 3D), and the recovery rate of first water flooding was 42–53%, which mainly sweeps the crude oil on the main channel (Figure 3B,C). The closer to the boundary, the more oil remains and the harder it is to reach (Figure 3D,G,J). In contrast, after injecting microorganisms, it could be observed that the distribution of crude oil tends to be heterogeneous after the second water flooding. The crude oil spread on both sides of the main channel has increased, and the remaining oil has significantly decreased (Figure 3E,H,K).

The calculation of oil recovery efficiency is shown in Table 1. The efficiency of second water flooding after microbial injection was positively correlated with the microbial injection amount and incubation time. The second water flooding of the control group was only able to increase the recovery rate by 8.69%. After the injection of 1 PV of microbial suspension, the sweep in the area near the main channel increased but the boundary was not obvious (Figure 3F), and the EORE(S) was 9.18%. After increasing the amount of injected culture to 2 PV, there was a significant driving on the crude oil at the boundary (Figure 3I). The main channel was not obvious and the crude oil tends to be homogeneous. The EORE(S) was 21.18%. Extending the incubation time to 6 days under the condition of maintaining 2 PV injection volumes, the EORE(S) increased significantly, by 44.77%. The main channel was almost island oil. The residual oil after the water flooding was evenly distributed and the boundary crude oil driving effects were obvious (Figure 3L).

### 3.2. Residual Oil in Different Regions after Second Water Flooding

**Figure 4:**
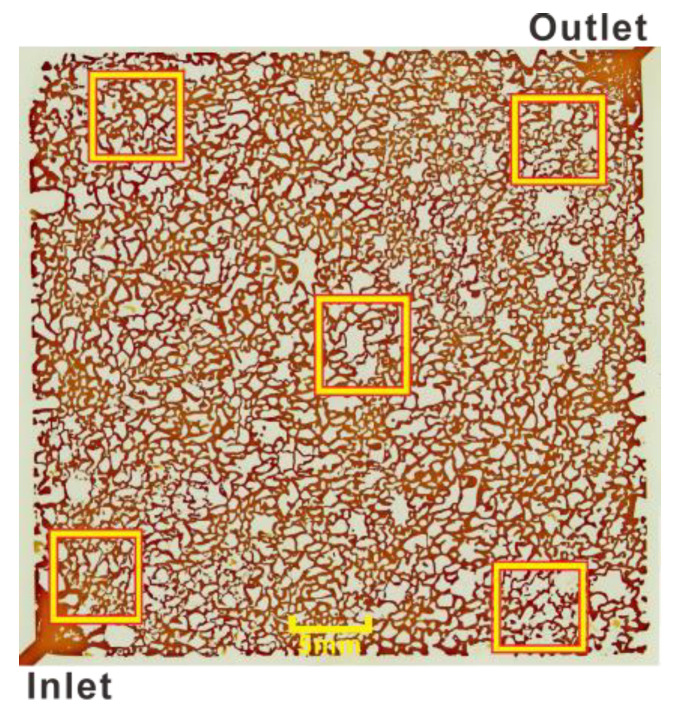
Residual oil observation windows.

**Figure 5:**
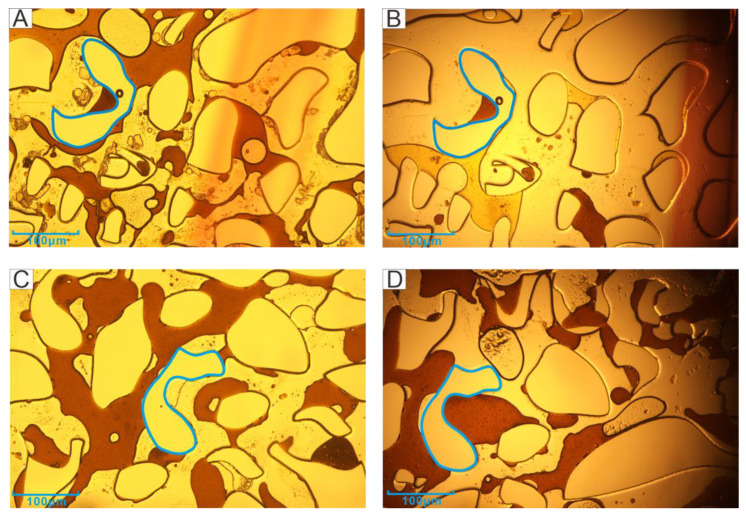
Accumulation state of residual oil in the port (**A**: the inlet after primary water flooding; **B**: the inlet after second water flooding; **C**: the outlet after primary water flooding; **D**: the outlet after second water flooding).

**Figure 6:**
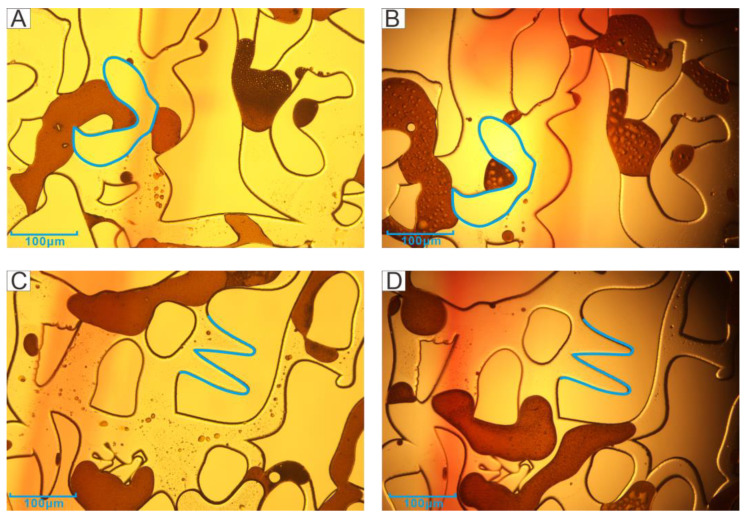
The residual oil status of the boundary after the second water flooding (**A**: Left boundary after the primary water flooding; **B**: Left boundary after the second water flooding; **C**: Right boundary after primary water flooding; **D**: Right boundary after the second water flooding).

**Figure 7:**
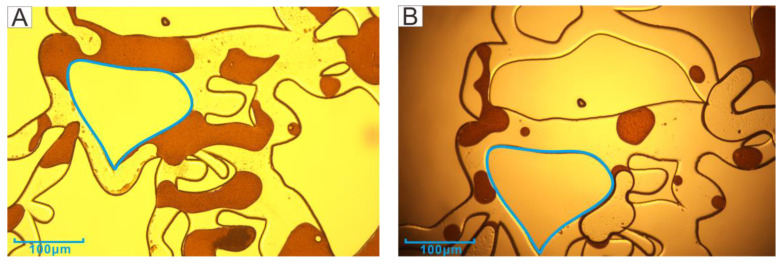
The residual oil status of the main channel (**A**—Status of channel after the first water flooding; **B**—Status of channel after the second water flooding).

Five location points in the model were selected as observation windows (Orange squares in Figure 4), located at the inlet (lower left), the upper boundary (upper left), the main channel (middle), the outlet (upper right), and the lower boundary (lower right). The blue areas in Figure 5, Figure 6, Figure 7 and Figure 13 were used as the positioning reference in the figures. The residual oil of the experimental groups and the control group were observed using a metallurgical microscope under 5× magnification (ShunYu CX40M, Ningbo, China). The remaining oil was mainly in the form of continuous sheets, oil films, clusters, and blind ends.

Figure 5 shows the comparison of the residual oil occurrence status after the primary (Figure 5A,C) and second (Figure 5B,D) water flooding. Most of the crude oil in the channel after the water drive at the inlet could be replaced, but there was still a lot of remaining oil, which was mainly in the form of continuous sheets and clusters. In addition, there was oil film on the channel wall and blind end oil (Figure 5A). After the second water flooding, it was obvious that the channel was almost clean, with continuous sheets and clusters oil stripped, and only the blind end crude oil remained (Figure 5B).

The residual oil at the outlet after the first water flooding was mainly in the form of continuous sheets, with a large number of residues and film-like oil remaining on the wall (Figure 5C). After injecting microorganisms, the continuous crude oil was emulsified and dispersed, mostly in clusters (Figure 5D). However, the displacement effect was insignificant compared with the inlet, and more crude oil remained.

Figure 6 shows the residual oil at the two boundaries. The left boundary was close to the inlet port. The channel was relatively clean after the second water flooding, but the residual oil still existed, mainly in the cluster as well as blind end oil. The remaining oil drive effect was not obvious. However, there were water droplets in the crude oil after the microbial action, forming the structure of water in oil (W/O) (Figure 6B), probably due to the biosurfactant promoting the oil–water phase solubility. The right boundary was the close to the outlet port. The more obvious phenomenon after microbial action was the formation of crude oil emulsion droplets, but the resulting droplets had larger particles (Figure 6D).

The main channel is located in the middle of the model, which is the location where the injected water and microorganisms have passed through for the longest time and were prioritized to stay. The residual oil in the channel after the first water flooding was mainly in the form of continuous flakes and isolated islands (Figure 7A). In contrast, the main channel after the microbial action only had some isolated oil and larger oil droplets, and the channel was very clean and water-wet (Figure 7B).

### 3.3. Petrology Characteristics

**Figure 8:**
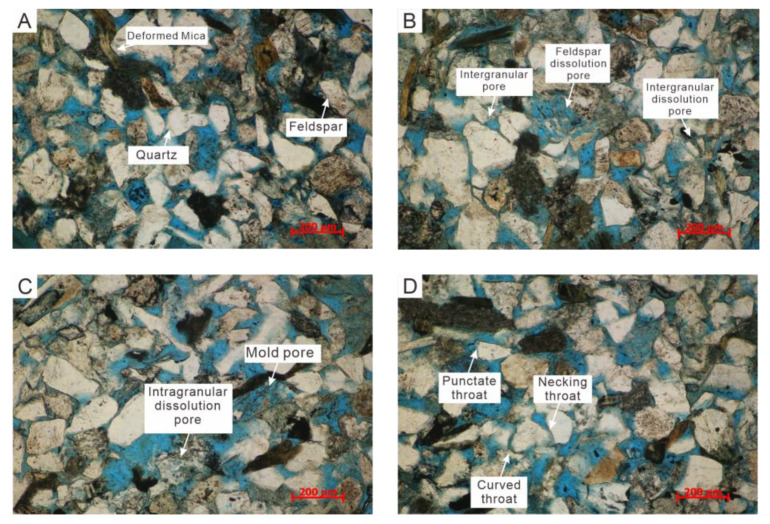
Microscopic pore and throat characteristics (**A**)—Rock debris; (**B**)—Pore type; (**C**)—Pore type; (**D**)—Throat type.

According to the results of casting thin sections, the four selected cores for this displacement are mainly fine-grained feldspathic sandstone (B-04 is fine-grained lithic feldspathic sandstone). The average content of debris is 87.87%. The average volume fraction of feldspar accounts for 40.88% of the debris content, while the average volume fraction of quartz is 45.13%. The rock debris is mainly composed of eruptive rocks with an average volume fraction of 5%, followed by quartzite rock debris with an average volume fraction of 2.75%. The filling material is mainly composed of clay (3%) and calcite (1.75%). Under the microscope, the deformation and bending of the mica could be seen (Figure 8A). The debris is mainly in point-line contact, and the bonding method is porous.

**Figure 9:**
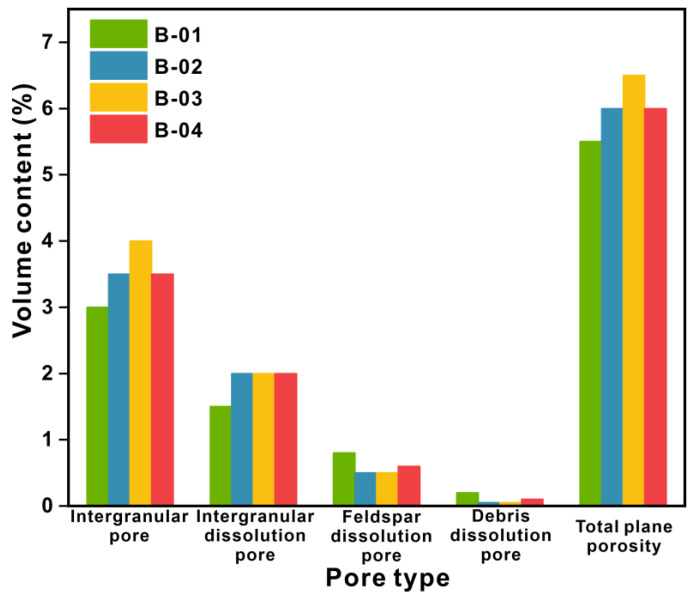
Pore type distribution.

The pore types of the four cores are mainly primary intergranular pores (3.5%) and secondary pores generated by dissolution, such as intergranular dissolution pore pores (1.88%), feldspar dissolution pores (0.6%), and rock debris dissolution pores (0.1%), with an average plane porosity of 6% (Figure 9).

The primary intergranular pores and secondary intergranular dissolution pores formed the main reservoir space (Figure 8B), which contributed the most to the storage space of the reservoir. In addition, feldspar dissolution pores and formed mold pores often increased pores, providing more storage space for oil and gas (Figure 8B,C). As for the type of pore combination, the samples had large pores and small throats. Combined with the observation of the cast thin section under the microscope, the throat was characterized by necking throat, punctate throat, and curved throat (Figure 8D). The punctate throat has large pores, and the throat is thick and short. The necking throat is usually formed by compaction and dissolution, which has large pores, but the throat is smaller than the point throat. The linear contact between fragments will form an elongated throat to connect pores and form a plated or curved throat. Those types of throat increase the flow resistance of the fluid and greatly reduce the permeability [34]. Therefore, although the samples had a large porosity, their connectivity was poor, and their permeability was low.

### 3.4. Characteristics of Pore and Throat

The lowest porosity of the four rock samples was 19.36%, and the highest was 21.06%. The lowest permeability was 0.26 mD, and the highest was 1.82 mD, with typical characteristics of high porosity and low permeability. The specific physical parameters are shown in Table 2.

**Figure 10:**
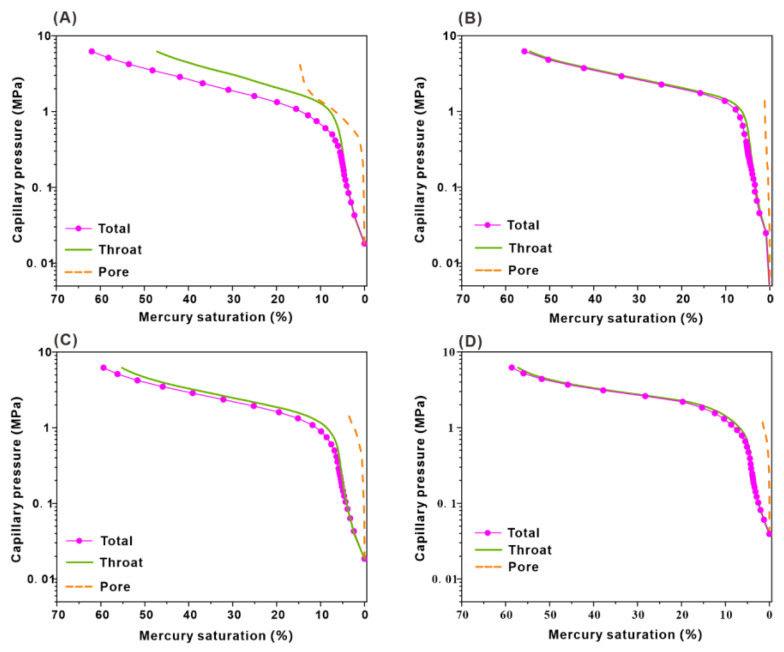
RCP capillary pressure curves of total, throat, and pore of samples (**A**: Core B-01; **B**: Core B-02; **C**: Core B-03; **D**: Core B-04).

The capillary pressure curves of the four samples have small differences (Figure 10). The morphology of the throat capillary pressure curve was basically consistent with that of the total capillary pressure curve. However, the pore capillary pressure curve was relatively steep, and the amount of mercury entering the pores was very small. The displacement pressure of sample B-01 was 0.29 MPa, the mercury saturation in the throat was 47.13%, and the mercury saturation in the pores was 14.80% (Figure 10A). The displacement pressure of sample B-02 was 0.5 MPa, the mercury saturation in the throat was 54.53% (Figure 10B), and the mercury saturation in the pores was 1.19%. The displacement pressure of sample B-03 was 0.42 MPa, the mercury saturation in the throat was 55.21%, and the mercury saturation in the pores was 4.18% (Figure 10C). The displacement pressure of sample B-04 was 0.78 MPa, the mercury saturation in the throat was 57.16%, and the mercury saturation in the pores was 1.42% (Figure 10D). The pore mercury saturation was much lower than that of the throat, indicating that the total mercury saturation of the four rock samples was controlled by the throat, and the reservoir mainly developed the micro throat.

**Figure 11:**
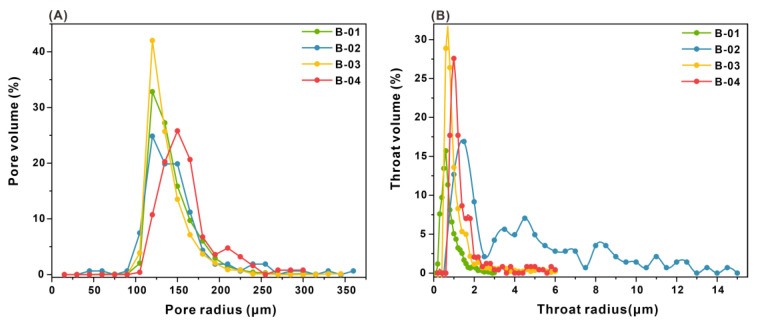
Distribution of pore (**A**) and throat (**B**) radius.

The histogram of pore radius distribution is shown in Figure 11A. It followed a normal distribution and exhibited a single peak. The pore radius was mainly distributed between 120 μm to 150 μm, with an average pore radius of 146 μm. The peak values of the four samples were close, 120 μm. The histogram of throat radius distribution is shown in Figure 11B. The throat radius of B-01 was mainly distributed between 0.4 and 0.8 μm and the average throat radius was 0.796 μm. The distribution range of the B-02 throat radius was relatively wide, with an average throat radius of 4.472 μm. The peak value was 1.5 μm. There is another small peak at 4.5 μm. This indicated an increase in the number of coarse throats in the sample [35]. The main throat radius distribution ranges of B-03 and B-04 were similar, ranging from 0.8 to 1.2 μm. The average throat radius of B-03 was 1.169 μm and 1.481 for B-04 μm.

### 3.5. Core Flooding Experiments

The volume of crude oil replaced by water flooding in core B-01 was 0.804 g, and the recovery rate was about 21.74%. After injecting 0.5 PV of microorganisms and soaking in the well for 3 days, water breakthrough occurred during the second water flooding, and a trace of crude oil was obtained. The fracture of the core was found when the gripper opened, which was presumed to be due to the shearing of the core caused by the uneven surrounding pressure. Core B-02 recovered 0.86 g of crude oil from the first water flooding, and the recovery rate was about 22.25%. After injecting 1 PV of microbial suspension and keeping well under 14 MPa pressure for 3 days, another 0.083 g crude oil was obtained. The microbial injection had a poor sweep effect. The first water flooding recovery of core B-03 was 0.856 g, with a recovery rate of 21.80%. When the soak time was extended to 6 days, the second water flooding recovery rate improved significantly to 6.98% (Table 3).

There is a significant difference in displacement efficiency between the real core and visual models. In order to explore the differences in mechanism of action, Core B-04 was directly injected with 1 PV of microorganism without first water flooding. The recovery of direct MEOR was 32.81%. Compared with direct water flooding (Cores B-01, B-02, and B-03), the recovery rate increased by more than 10%. The second water flooding efficiency rate was 2.69%. 

## 4. Discussion

Both microscopic visual models and real core displacement experiments confirmed that the injection of microbial fluids after conventional water drives has a secondary improvement in recovery efficiency. Studies at both scales also confirmed a positive correlation between displacement efficiency and microbial injection and cultivation time. The improvement in oil recovery efficiency was mainly due to the increase in oil washing efficiency and sweep efficiency [23].

### 4.1. Effect of Emulsification on Recovery Efficiency

The single strains in the consortium bacteria (B-ALL) had been proven to produce glycolipids and lipopeptides [36]. These biosurfactants had a strong emulsification ability. Oil droplets would be formed by the action of biosurfactants. On the other hand, due to the special amphiphilic nature of surfactants, they also had the effect of changing channel wettability and reducing interfacial tension [6,13,14,37], and the above methods could effectively improve the recovery rate [38,39].

**Figure 12:**
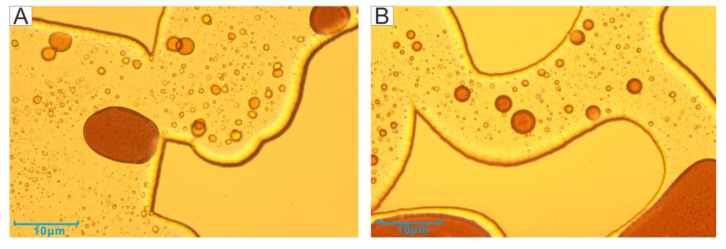
Oil droplets formed by emulsification (**A**)—change of wettability; (**B**)—oil droplets in the channel.

Therefore, with the increase in injection volume, the content of biosurfactant also increased, which was more favorable to the formation of oil droplets. The oil droplets, which were formed after 6 days of incubation with 2 PV of microorganisms injected under the microscope (50x magnification), were shown in Figure 8. The oil droplets formed were distributed within the channel (Figure 12B), while the oil droplets near the well bore were not in contact with the well bore, and the contact angle was very small (Figure 12A). In addition, the second water flooding also removed most of the oil film in the channel, and the cleaner channel wall (Figure 5B) indicated a change in wettability and a reduction in adhesion work [40,41,42]. In core experiments, B-04 is directly injected with microorganisms. The direct contact between microorganisms and crude oil results in an increase in extraction rate of over 10% compared with other cores, which was also due to the emulsification. 

**Figure 13:**
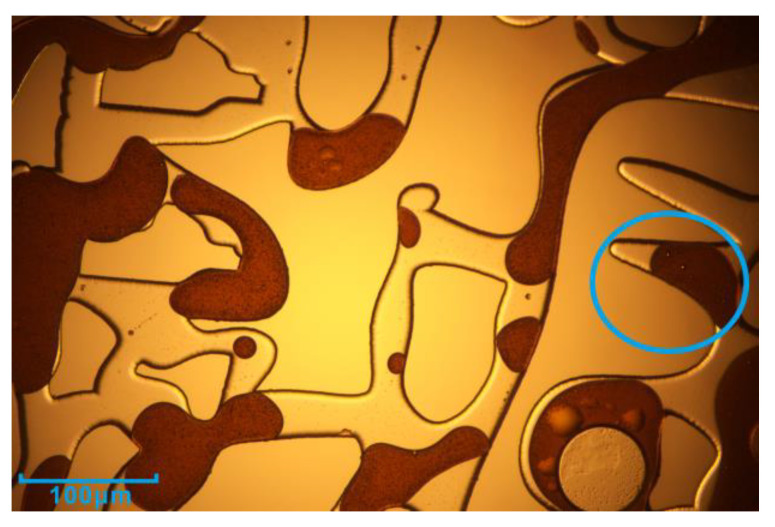
The sweep of the blind end.

The phenomenon of water in oil at the boundary is also the performance of emulsification. Xu et al. [43] showed that this water-in-oil emulsion has a certain effect on the blind end, like ‘miscible flooding’, and there is no capillary. The initiation of the blind end was also observed in this study (The blue circle in Figure 13).

### 4.2. The Change of Wave Efficiency

In the visualization experiments, the distribution of crude oil in the channel after the secondary water flooding all tends to be heterogeneous, and the boundary crude oil drive was obvious (Figure 3F,I,L). This indicates the improvement of wave efficiency. Many studies have shown that the oil droplets have the effects of clogging the pore, allowing fluids to flow vertically, from the high permeability layer to the middle and low permeability layer [44,45,46]. However, these large oil droplets are thermodynamically unstable and will be separated into two phases [47]

Although an increase in the sweep was observed in the microscopic experiments as well as a significant increase in the recovery efficiency (Table 1), the highest second water flooding recovery efficiency in the core flooding was only 6.98%, which was much smaller than in the microscopic experiments. The radius difference between pores and throats was significant, making them typical of large pores and small throats. This was also characteristic of tight sandstone reservoirs, where the reservoir space was composed of large pores and small throats, with strong heterogeneity [15]. The strong heterogeneity of pore throat is an important factor causing a significant decrease in permeability. On the other hand, the compaction and pressure dissolution between debris particles, as well as the development of necked and curved throats, greatly reduced the connectivity between pores, resulting in lower permeability. Affected by the “bottleneck” effect, large pores and small throats cannot produce high permeability, which was also the particularity of tight sandstone.

Microscopic models are mostly highly porous and have comparatively highly permeable channels, so microbial transport in models was relatively easy. On the other hand, it has been pointed out that the emulsion droplet size has some influence on mobility [48]. Therefore, microorganisms need a longer time to function in the pore space. This is also the reason for the significant improvement in recovery efficiency (from 2.69% to 6.98%) after the extended soak time.

### 4.3. The Potential Environmental Impact of Consortium Bacteria

The MEOR method is considered a green and environmentally friendly recovery method, mainly due to its metabolites and products causing less damage to reservoirs and equipment [13,49]. The consortium bacteria used in our research are all *Bacillus* and the main metabolic product were lipopeptides. Based on previous research, the microbial culture medium is mainly alkaline [24], avoiding the generation of acidic substances and corrosion of pipelines. Besides, in order to save costs, oil fields generally implement reinjection of produced water, which has high salinity and is accompanied by sludge deposition [50]. Consortium B-ALL could adapt to high salinity environments, and according to existing research, petroleum-degrading bacteria can act on oil spillage, oil sludge, and bioremediation [10,49].

In addition, when injecting consortium bacteria on site, there will be competition with the in situ microbial community [51]. However, the bacteria used in this study were screened from the same layer, which is not prone to exclusion, but may alter the community structure in the reservoir. Related research will be conducted in further on-site applications, focusing on the changes in microbial communities that have negative effects on reservoirs such as sulfate-reducing bacteria, and it will further explore the environmental impact of B-ALL injection.

## 5. Conclusions

Both microscopic and core oil flooding tests have verified the effectiveness of the consortium bacteria on enhanced recovery. The microbial injection volume and cultivation time were positively correlated with recovery efficiency. The micro visualization model with high porosity and permeability, after injecting 2PV microbial suspension and soaking for 6 days, showed that the secondary water drive extraction efficiency can reach 44.77%. The microorganisms in the channel which produce biosurfactants to emulsify crude oil, combined with degradation, can increase the oil washing efficiency. In addition, large oil droplets also had the effect of clogging the pore space and improving the wave efficiency. For tight reservoirs, the maximum recovery rate was 6.98%, while for high porosity and permeability reservoirs, the recovery rate was 44.77%. Microorganisms in the channel mainly emulsify the crude oil by biosurfactants and change the wettability to achieve the oil washing effect. This study validates the role of microorganisms in enhancing recovery and provides a basis for the application of MEOR in real oil fields. In the future, we will optimize the industrial fermentation process of consortium bacteria, making large-scale industrial production and applying it on-site.

## Figures and Tables

**Table 1:** Micro model oil recovery efficiency.

Sample	Initial Crude Oil %	EORE * (W)%	EORE * (M)%	EORE * (S)%
Control	30.27	42.98	/	8.69
1PV 3D	26.49	53.82	5.65	9.18
2PV 3D	34.91	52.07	27.04	21.18
2PV 6D	34.91	45.57	3.97	44.77

* EORE: Enhanced oil recovery efficiency.

**Table 2:** RCP data of the samples.

No.	Porosity (%)	Permeability (mD)	Average Pore-Throat Radius Ratio	Displacement Pressure (MPa)
B-01	21.06	1.82	259.85	0.29
B-02	19.36	0.26	66.98	0.50
B-03	20.47	0.75	169.08	0.42
B-04	19.41	0.35	143.86	0.78

**Table 3:** Core flooding recovery efficiency.

No.	W_S_ (g)	Oil Weight of Water Flooding (g)	Efficiency of Water Flooding %	Oil Weight of Second Water Flooding (g)	Efficiency of Second Water Flooding %
B-01	3.698	0.804	21.74	Trace	/
B-02	3.866	0.86	22.25	0.083	2.15
B-03	3.926	0.856	21.8	0.258	6.98
No.	Saturated oil quality (g)	Oil weight of MEOR	Efficiency of MEOR	Oil weight of second water flooding (g)	Efficiency of second water flooding %
B-04	3.974	1.304	32.81	0.107	2.69

## Data Availability

The geological and geochemical data used to support the findings of this study are included in the article.
